# Anæsthetics for Dental Operations

**Published:** 1869-11

**Authors:** Walter Bruce


*To be Continued.*

---

### ARTICLE II.

### *Anæsthetics for Dental Operations.*

### Ether, Chloroform and Nitrous Oxide.

By Walter Bruce, D.D.S.

The agents commonly used to produce anæsthesia are ether, chloroform and nitrous oxide, the latter being particularly interesting to us as dental surgeons, since so far as its merits up to this time have been tested it answers the purposes of a dental anæsthetic better than any other that has yet come into the possession of the profession. There are other *local* anæsthetics, the best of which are ether spray and rhigoline spray, which may be sometimes advantageously used in cases where general anæsthetics are contra-indicated, and where it is desirable more to overcome the reluctance of the patient than to produce any decided anæsthetic effect. The honor of the discovery of anæsthesia or of producing insensibility to pain during surgical operations by means of certain agents, is properly due to American dentists. Dr. Horace Wells. of Hartford, Connecticut, induced anæsthesia with nitrous oxide in 1844, and Dr. Morton, of Boston, Massachusetts, discovered the anæsthetic property of ether. He performed the first painless operation (extraction of a tooth), rendered so by the anæsthetic property of ether on the 30th, of September, 1846. During the following month —October—two more operations were performed with ether —one the excision of a tumor on a man's face, and the other the excision of a fatty tumor on the arm of a female. These operations were performed by Drs. Warren and Heywood, of the Massachusetts General Hospital. In the first operation the anæsthesia was not complete, it being difficult to keep up insensibility whilst operating on the face ; in the second it was. On the 7th of November, at the same Hospital, Dr. Morton administered ether to a female upon whom was performed the operation of amputation at lower third of thigh for diseased knee joint. In this case the anæsthesia was complete, the operation having been performed without any knowledge of it at the time on the part of the patient The administration of ether by inhalation had been long known and used with very beneficial results by some physicians as an expectorant. More than a century ago, Dr. Pearson, of London, was in the habit of administering as an expectorant an ethereal tincture of cicuta leaves, with which he sometimes partially produced anæsthesia. It is also thought by some, that the celebrated John Baptista Porta, author of Natural Magic, produced his anæsthetic effects or insensibility by means of ether inhalation. A good many years ago, in the early part of this century, the boys in a part of the city of Philadelphia, known as Northern Liberties, were in the habit, merely for sport, of inhaling ether. The effects were various, from a slight exhilaration to profound stupor. A death from meningitis having occurred as a consequence of this sport, the municipal authorities put a stop to it. A number of other cases of insensibility produced by ether are mentioned in different medical journals. But, though anæsthesia was hinted at, and near being discovered by different individals at different times, it has remained for the present generation and a member of the young profession of dentistry to demonstrate its practicability and applicability in surgery. The medical profession has long been acquainted with a liquid possessing the properties of sulphuric ether. The name ether—spiritus ethereus—was first given to it by a German chemist named Froben, who lived in the early part of the eighteenth century. Ether is the oxide of a compound radicle called ethyle. Its formula is $C_4H_5O$, and differs from alcohol only by the proportions of water; alcohol being composed of $C_4H_6O_2$. It is one of the products of the destructive distillation of sulphovinic acid, or, what is equivalent, equal weights of strong alcohol and oil of vitriol. Sulphovinic acid is the bisulphovinate of ether, its formula being $C_4H_5O2SO_3HO$. By means of the heat applied in distilling, the ether ($C_4H_5O$) is liberated, passes over and is condensed in the receiver. In order to purify it, it is mixed with caustic potassa and gently redistilled. A proper regulation of the amount of heat, and other precautions are necessary in conducting the distillation and purification. Ether is a clear, colorless, transparent, thin liquid, of a peculiar penetrating odor, at first, to many persons not unpleasant, and of a hot, pungent taste, followed by a sensation of coolness in the mouth. According to the United States Pharmacopoeia it has a specific gravity of 0, 750, and boils at a temperature of 96° Fahrenheit under ordinary atmospheric pressure. When dropped on the hand it leaves a sensation of coolness, caused by the rapid evaporation. It is very volatile and highly inflammable. When pure and recently prepared it is neutral, but when exposed to the atmosphere and light, it absorbs oxygen and becomes acid by the production of acetic acid and water. It is miscible with alcohol in all proportions, but one part of ether requires ten parts of water to dissolve it. It dissolves volatile oils and some of the fatty and resinous substances. Taken internally in the liquid form, it is a diffusable excitant; is also excitant if inhaled in moderate quantity. In large doses it acts like alcohol somewhat, producing intoxication; in still larger doses narcosis. It can be detected, like alcohol, in the pulmonary exhalations, and the brain of those who have died after it has been largely exhibited. The impurities to which ether is liable are acids, water,alcohol and heavy oil of wine. The acids may be detected by litmus paper. To detect the water a very good way is to agitate in a minim measure a quantity of ether with half its volume of concentrated solution of chloride of calcium. The chloride of calcium takes up the water in the ether, and the diminished bulk indicated on the graduate measure will show the amount of water that was present in the ether. Alcohol can be detected, unless it be present in very large amount, by mixing a portion of the suspected ether with water; the alcohol leaving the ether and uniting with the water, two layers or strata of liquid being formed. If this be done in a graduate measure the amount of alcohol present can be easily seen. The presence of heavy oil of wine is shown by the ether becoming milky when dropped in water. Any fixed oils or solid substances held in solution can be detected by evaporating a portion of the ether. Ether to be pure ought to leave no taste or odor when evaporated on the palm of the hand. A good test for the purity of any article of ether is to find its specific gravity, all other articles with which it is usually adulterated having a tendency to increase its density. The United States Dispensatory says, that a small quantity of water is admissable in officinal ether, but for anæsthetic purposes it should always be as pure as possible.

The next article to noticed for the production of anæthesia, and one which has to a great extent superseded ether, is chloroform. It was discovered in the year 1831 simultaneously by Mr. Guthrie, of New York and Soubeiran, of Paris. Several years afterwards, Dumas determined its elementary composition and gave it the name of chloroform. It was used as a diffusible stimulant in medical practice, and in many diseases as a substitute for alcoholic liquors. Dr. Dunglison classifies it under the head of narcotics, and according to Dr. Hartshorne a fluid drachm of chloroform is equal in hypnotic effect to thirty or thirty-five drops of laudanum. Its anæsthetic property was discovered by Prof. Simpson, of Edinburg, in 1846. Prof. Simpson had been experimenting for some time with anæsthetic agents in order to discover a better one than ether. His attention was called to chloroform for that purpose, by a gentleman in Liverpool, and upon trial it proved a success. Shortly after this discovery it was put to public test by anæsthetizing with with it a patient, upon whom Dr. Miller, Professor of Surgery, operated for necrosis of radius. After this its fame and use soon spread throughout Europe, but has not yet entirely supplanted the use of ether in this country. Leibig calls chloroform a perchloride of formyl. Formyl is a compound radicle composed of $C_2H_1$. Chloroform may be expressed $C_2H_1Cl_3$. If, by any means, the three parts of chlorine be exchanged for three parts of oxygen the chloroform becomes converted into formic acid. Chloroform is manufactured on a large scale by carefully distilling together good commercial chloride of lime, alcohol and water. The following is Dumas' formula: Chloride of lime in powder, 4 pounds, water 12 pounds, rectified spirits 12 fluid ounces. This is to be distilled and purified according to specific directions. Chloroform is a clear, colorless, limpid fluid, with a bland, ethereal, fragrant, fruit like odor. Its taste is at first sweetish, afterwards becoming hot and pungent. It is easily dissolved in alcohol or ether, but very sparingly soluble in water; according to some authorities requiring 288 times its bulk of water to dissolve it; it dissolves wax, resin, gutta percha, caoutchouc, comphor and other substances; it is inflammable; its vapor, however, burns with a greenish, smoky flame, and it renders the flame of an alcohol lamp yellow and fuliginous. It evaporates easily, though not so easily as ether. The elevation of the temperature greatly increases its vaporization. It boils at 141° Fahrenheit, and has a specific gravity of 1.49 according to the U. S. Pharmacopoeia. When pure it has no action on potassium, but an alcoholic solution of caustic potassa decomposes it, producing chloride of potassium and formate of potassa. According to Dr. Dunglison, a pure article applied locally does not irritate the skin, but alcohol renders it caustic. According to Dr. Stille it has, when applied locally, considerable counter irritant effect and also anodyne. When rubbed on the palms of the hands it quickly evaporates leaving scarcely any odor. Chloroform is liable to contain alcohol, ether, and chlorinated pyrogenous oils. The admixture of ether is not of any great moment, a mixture of this sort called chloric ether, being frequently used, and by some preferred, for producing anæsthesia, but the admixture of alcohol with the chloroform makes it vesicant to the skin and irritating to the mucous membrane of the air passages. Both alcohol and ether lessen the specific gravity of chloroform, and Soubeiran recommends, in order to detect the presence of substances which have this effect, to add a drop of the suspected liquid to equal parts of concentrated sulphuric acid water. The specific gravity of such a liquid, when cold, is 1.38, and chloroform being of greater density will sink in it. M. Miahle has proposed the following for the presence of alcohol: Add a drop of the chloroform to distilled water, if pure it will sink to the bottom of the glass and remain there transparent, but if the slightest quantity of alcohol be present the globules assume a milky appearance. Of all the impurities, however, to which chloroform is liable, the most injurious are the pyrogenous oils. When inhaled, or sometimes even smelt, they produce distressing headache and nausea. The test for these oils is pure concentrated sulphuric acid. Pure chloroform mixed with an equal volume of this acid does not color it, but if any of these oils be present the color varies from yellow to redish brown, according to the amount of impurities. If chloroform containing these oils be allowed to evaporate on the hand, they will remain and may be recognized by their their peculiar offensive smell. Pure chloroform evaporates on white paper without leaving any taste. Anæsthesia is produced with ether and chloroform by the inhalation of their vapors. They affect the animal economy very similarly and are administered in the same manner. Ether is said to be less dangerous than chloroform, the effects being more transient, and the after effects not so prolonged, owing probably to its being more quickly volatized and more easily eliminated from the system. Some think that the different chemical constituency of chloroform account for its greater potency and more decided toxical effect upon the system. Whatever may be the cause of the difference between the two agents, certainly more deaths have taken place under the use of chloroform than under ether. The former is more generally used and being a more potent article is more apt to be attended with fatal consequences in the hands of those who do not thoroughly understand its administration. A number of experiments on animals have been made by different men of science with these agents. There is some discrepancy on some points in regard to their results but in the main they coincide. If ether be administered to an animal—rabbit—it manifests, at first, feelings of distress, tries to escape; these phenomena are soon succeeded by a condition of excitement, increased action of the heart and arteries, excited condition of the nervous centres and quickened respiration; a stage of depression now commences, lessening of the frequency of the action of the heart, and of respiration, impairment of muscular action; the animal staggers, falls upon its side, the sphinctors relax, attended sometimes with voiding of the urine and fœces, complete insensibility and muscular relaxation. If air be now admitted the animal soon revives, but if the administration of the vapor be continued, the pulsations and breathings rapidly diminish, there are symptoms of cerebral congestion, cold extremities, lividity of the lips. We have the symptoms of complete coma and finally cessation of the action of the respiratory organs and of the heart. According to the investigations of M. Flourens and others the nervous centrés appear to be brought under the influence of these anæsthetics in the following order: First the cerebrum, the seat of the intellectual faculties, next the cerebellum, the seat of the power of co-ordinating muscular movements, then the spinal cord with its reflex or sensori-motor functions, and lastly, if the agents be pushed far enough, the medulla oblongata or

"respiratory tract," resulting in a cessation of, or rather as a necessary consequence of this, stopping of the respiration and death. The vapor of ether or chloroform then, administered to a certain extent, produces insensibility attended with either complete or incomplete loss of consciousness; if carried far enough beyond this extent, death is the result. Loss of sensibility takes place before loss of motion. If during the administration of the vapor it be diluted by admixture with air, the symptoms can be more gradually produced and prolonged to a greater length of time. During the inhalation the arterial blood becomes dark, and according to some authorities insensibility is produced before any dark blood can be detected in the arteries. Upon the withdrawal of the anæsthetic and the admission of air to the lungs, the arterial blood assumes its brighter normal hue. In blood drawn from an anæsthetized person the amount of the fibrine is smaller and the clot is more condensed, whilst there is an increase in the amount of the serum. The blood of horses, to which these agents had been administered, retained the ethereal odor for several days afterwards, and a goat retained the ethereal odor in its milk for five days. Dogs have been restored by depletion even when the agents had been given until they ceased to breathe. The amount of fat in the brain was diminished by the administration of these agents according to Van Bibra, and increased in the liver. If animals are killed during the first stage of the anæsthesia the lungs are found very much congested, in the second stage they are frequently found emphysematous. If either of these agents be taken into the stomach in the liquid form it produces intoxication; and chloroform in large doses, an ounce or more, almost always results in death, the autopsies revealing inflammation of the stomach, sometimes attended with a softened condition of the mucous membrane of the pharynx and air passages, and congestion of the brain. The Russian Surgeon, Pirogoff, has shown by experiments on animals that if the vapor of ether or chloroform be injected into the rectum, or if the liquid be slowly injected the same anæsthetic effect is produced as by inhalation. Ether and chloroform are both administered in the same manner. A number of inhaling bottles and apparatuses have been invented for the more easy introduction of the vapor. The simplest and best means for this purpose is a napkin or towel folded into a funnel shape around the hand, and the ether or chloroform dropped into the bottom of this, or else a cone shaped piece of tin or paste-board, in the bottom or apex of which is a sponge on which the agent is poured. Some prefer a handkerchief moistened with the anæsthetic fluid applied to the mouth and nostrils. Others again prefer simply a single layer of cotton cloth laid over the mouth and nose on which the agent is gradually dropped. This latter is Prof. Simpson's method. The patient to be anæsthetized is placed in the recumbent posture, the "decubitus dorsalis," on a couch, the head slighly raised by a pillow, and, if the weather will permit, the patient should be in the open air, or else in a large airy room where the windows can be raised and currents of fresh air freely admitted, should it become necessary. All parts of the dress should be loosened, so as to permit free play of the diaphragm and other muscles of respiration, and allow unimpeded circulation of the blood. If the patient be feeble or timid it would be advisable to precede the inhalation with brandy or brandy and laudanum. The administration of brandy before inhalation was considered always safe practice by the Confederate Surgeons during the war, and was rarely ever omitted when the article was to be had. The mouth and nose should now be anointed with glycerine or oil to prevent any vesicating effect of the chloroform, if that be the agent administered, and a few words of comfort and assurance addressed to the patient. The folded napkin with the ether or chloroform in it, is brought near the face. It should be made to approach gradually for fear of producing too great suffocating effect, and should not be allowed to come nearer than one inch of the face, so as to allow admixture of air with the vapor. By observing these. precautions the anæsthesia is produced more gradually and with less risk to the patient, enabling us to observe the successive changes as they occur, and at any stage of the administration comprehend the condition of the patient. Silence should be enjoined upon the by-standers, and in cases where we are doubtful of the propriety of administering, and indeed in all cases, it would not be amiss to have at hand the means for effecting resuscitation. The patient should not be raised during the inhaling or anæsthesia, for fear of producing syncope. Considerable tact is sometimes required in the administration. The symptoms or condition of the patient is to guide us in regard to the amount of vapor required and its adjustment or proportionment. The first effect of the inhalation upon the system is to increase vascular action, the pulse becomes quicker, the heart beats with more frequency and the blood is sent with greater force to the surface. The face is slightly flushed, the countenance assumes a more lively expression, the breathings quicken and the skin grows moist and warm.

*To be Continued.*

---

